# The Association of Combined Per- and Polyfluoroalkyl Substances and Metals with Allostatic Load Using Bayesian Kernel Machine Regression

**DOI:** 10.3390/diseases11010052

**Published:** 2023-03-22

**Authors:** Tahir Bashir, Emmanuel Obeng-Gyasi

**Affiliations:** 1 Department of Built Environment, North Carolina A&T State University, Greensboro, NC 27411, USA; 2 Environmental Health and Disease Laboratory, North Carolina A&T State University, Greensboro, NC 27411, USA

**Keywords:** PFAS, metals, allostatic load, BKMR, mixtures

## Abstract

*Background/Objective:* This study aimed to investigate the effect of exposure to per- and polyfluoroalkyl substances (PFAS), a class of organic compounds utilized in commercial and industrial applications, on allostatic load (AL), a measure of chronic stress. PFAS, such as perfluorodecanoic acid (PFDE), perfluorononanoic acid (PFNA), perfluorooctane sulfonic acid (PFOS), perfluoroundecanoic acid (PFUA), perfluorooctanoic acid (PFOA), and perfluorohexane sulfonic acid (PFHS), and metals, such as mercury (Hg), barium (Ba), cadmium (Cd), cobalt (Co), cesium (Cs), molybdenum (Mo), lead (Pb), antimony (Sb), thallium (TI), tungsten (W), and uranium (U) were investigated. This research was performed to explore the effects of combined exposure to PFAS and metals on AL, which may be a disease mediator. *Methods*: Data from the National Health and Nutrition Examination Survey (NHANES) from 2007 to 2014 were used to conduct this study on persons aged 20 years and older. A cumulative index of 10 biomarkers from the cardiovascular, inflammatory, and metabolic systems was used to calculate AL out of 10. If the overall index was ≥ 3, an individual was considered to be chronically stressed (in a state of AL). In order to assess the dose-response connections between mixtures and outcomes and to limit the effects of multicollinearity and other potential interaction effects between exposures, Bayesian kernel machine regression (BKMR) was used. *Results*: The most significant positive trend between mixed PFAS and metal exposure and AL was revealed by combined exposure to cesium, molybdenum, PFHS, PFNA, and mercury (posterior inclusion probabilities, PIP = 1, 1, 0.854, 0.824, and 0.807, respectively). *Conclusions*: Combined exposure to metals and PFAS increases the likelihood of being in a state of AL.

## 1. Introduction

### 1.1. Background

The totality of exposures people endure throughout their lives and how those exposures affect health have been referred to as the exposome [1]. Although certain environmental exposures might lead to unfavorable health outcomes, little is understood about how these factors interact or synergize to affect the stress response system [2]. This is especially critical to understand when exposure to metals is mixed with exposure to per- and polyfluoroalkyl substances (PFAS).

The negative consequences of PFAS and mixed metals may be deleterious. They could have a long-term effect on the impacted populations’ social, educational, and economic advancement [3,4]. In many environments with high levels of chronic stress, several metals and PFAS co-exist at moderate to high levels.

Individuals maintain physiological balance through allostasis, which involves adjusting bodily characteristics to meet environmental requirements. Homeostasis describes health as a state in which all physiological parameters function within non-changing setpoints. Allostasis, on the other hand, states that there are no setpoints and that the demands of the moment will determine the normal values of markers. However, the body adjusts to the higher set point if the impediments persist [5]. When the setpoint is changed, people are said to be in a state of allostatic load. Allostatic load (AL), an index of persistent physiological stress, is the biological consequence of stress. AL depends on the assumption that repetitive activation of the hypothalamic-pituitary-adrenal (HPA) axis affects multiple organ systems [5,6,7,8].

The wear and tear on the body caused by ongoing exposure to stressors can be measured by AL, which combines markers from systems within the human body to form a comprehensive biological stress index. An adult’s well-being is negatively impacted by psychosocial stresses, such as poverty, racial inequality, lack of access to resources, and water and food insecurity, which may be combined with environmental factors to increase AL within populations.

At the individual and population levels, real-world human exposure to stressors is extraordinarily varied and temporally dynamic. Humans are constantly exposed to intricate chemical combinations of PFAS, metals, and other environmental pollutants [9,10]. Data analytics techniques provide a novel way to analyze the combined risk of various exposures in order to develop methodologies to properly identify and evaluate their impact on indices of stress, such as AL, because we do not fully understand the combinational nature of these exposures [11].

### 1.2. Human Exposure Pathways to PFAS and Metals

According to the Agency for Toxic Substances and Disease Registry at the Centers for Disease Control and Prevention (CDC), metals such as cadmium (Cd), arsenic (As), lead (Pb), and mercury (Hg) are among the top 10 most toxic substances. Most people are exposed to metals through ingestion (through water and food), inhalation (through cigarette smoke or industrial products), or skin contact (through paint or soil) [12]. For example, As comes in two forms: the inorganic form is highly toxic, while the organic form is not. Most people are exposed to inorganic As, which is found in soil and groundwater, through drinking water, often from unregulated private wells. Most people are exposed to organic arsenic, which is found in fish and shellfish, through ingestion [13]. In the United States, people of different races, ethnicities, and socioeconomic backgrounds experience widely varying degrees of exposure. For example, non-Hispanic blacks have higher Pb exposure than non-Hispanic whites [14].

Humans most commonly absorb toxic PFAS through their diets [15]. Inhalation of air or dust containing PFAS particles is another route of exposure. Over the past decade, there has been extensive research on the dangers of PFAS exposure for people’s health. The CDC, for example, has set limits on PFAS concentrations in drinking water (70 ppt for PFOA and PFOS).

PFAS spreads through many sites, including landfills and sites where PFAS has been processed. E-waste sites, for example, leach PFAS into groundwater, soil, and air, while wastewater treatment plants (WWTPs) release PFAS-laden effluent into rivers, lakes, and farms [16]. PFAS from treated or untreated effluent enters sewers, rivers, lakes, and oceans through aquatic ecosystems, making water the ultimate repository of PFAS in the environment [2].

Pregnant and parturient women, elderly people, children, and neonates are the most vulnerable to PFAS exposure, which can cause thyroid, lung, kidney, reproductive organ, metabolic, brain, and behavior disorders, obesity, type 2 diabetes, proteinuria, hematuria, immunosuppression, and adverse pregnancy outcomes [17].

### 1.3. Bayesian Kernel Machine Regression (BKMR): A Mechanism for Monitoring Multiple Environmental Exposures

Bobb et al. introduced Bayesian kernel machine regression (BKMR) for analyzing mixtures within the R statistical program [18]. By using the (bkmr) package for the R programming language, BKMR was created to estimate the health effects of pollutant mixtures and is used for toxicological, epidemiological, and other applications. It does this by using procedures from Gaussian predictive methods or hierarchical variable selection [18,19]. The estimation of health outcomes of the mixtures under kernel function is modeled on the exposure variables by adjusting for potential covariates or cofounder factors [20]. These procedures can address the possible collinearity of the mixtures’ components and test the exposures’ overall health effects [21].

Ultimately, BKMR modeling is a technique that (1) models the exposures and outcomes comprehensively, (2) evaluates the components of chemicals independently of the independent–dependent function, (3) evaluates the effects of mixtures of chemicals, and (4) distinguishes the necessary chemical mixtures for any dataset that is simulated [19,21]. BKMR is also used to solve the challenges encountered when evaluating the health impacts of chemical mixtures (i.e., PFAS and metals). In epidemiological and toxicological studies, BKMR helps solve problems such as collinearity and strong correlations between exposures [22]. BKMR uses variable selection that produces and estimates posterior inclusion probabilities (PIPs) values, which measure the values of variable importance for each exposure in a mixture [18,20].

This study using BKMR hypothesizes that exposure to metals and PFAS is associated with high levels of AL. PFAS and metals were chosen due to the unique opportunity to assess combined exposures to organic and inorganic contaminants, the extensive research on both groups of contaminants with National Health and Nutrition Examination Survey (NHANES) data, and the vast historical and emerging research related to these contaminants. To test this hypothesis, data from the NHANES were used to identify the factors most critical in combined exposures to PFAS and metals.

## 2. Materials and Methods

### 2.1. Study Cohort and Design

Data from the NHANES 2007–2014 of adults aged 20 years and over were utilized in this investigation. This dataset is a representative sample of non-institutionalized people residing in all 50 U.S. states and the District of Columbia. The U.S. Centers for Disease Control and Prevention (CDC) collected the data, which are available in two-year cycles and include multi-year, stratified, multi-stage, and clustered samples. The population of the United States is represented by the statistics for four cycles within 2007–2014.

Selected individuals in the NHANES underwent a physical examination and an interview. The participants’ blood was extracted, and samples were sent to a laboratory for evaluation.

On the NHANES website of the CDC, additional descriptions and information about the study, as well as the steps and processes involved in data collection, are provided.

The association between the various PFAS/metals concentrations and AL levels was examined using weighted data in order to produce sample estimates, which reflect how many people in the U.S. population one individual represents.

### 2.2. PFAS and Metals Measurements

There were two examination sessions each day. Exams in the morning, afternoon, or evening were randomly assigned to participants. After fasting for nine hours, participants were instructed to consume 75 g of dextrose (10 oz. of glucose solution) within 10 min after the initial blood draw. After the first blood draw was taken, a second blood sample was taken [23].

#### 2.2.1. PFAS Quantification

At the mobile examination center (MEC), the CDC gathered blood samples for laboratory analysis to evaluate serum for PFAS. Polypropylene or polyethylene containers were used to store the serum samples. The vials were subsequently shipped to several laboratories across the country. Sample analysis was performed at every survey location under the same conditions, owing to the controlled environments at separate facilities.

In order to concentrate the analytes (PFAS) in a solid-phase extraction column, one aliquot of 50 mL of serum was injected into a commercial column switching system after being diluted with formic acid.

High-performance liquid chromatography was used to separate the analytes from one another and the other serum constituents.

A negative-ion Turbo Ion Spray (TIS) ionization source was utilized for detection and quantification (DOQ). Tandem mass spectrometry was used to change liquid-phase ions into gas-phase ions, utilizing a variation of the electrospray ionization source.

These PFAS can be quickly detected in human serum using this technique, with detection limits in the low parts per billion (ppb or ng/mL) range [24].

An imputed value was placed in the analyte results field for analytes with analytic results below the lower limit of detection; 0.10/square root of 2 = 0.07 was the lower limit of detection divided by the square root of 2. Thus, the LOD for each PFAS was 0.10 or 0.07.

#### 2.2.2. Metals Quantification

Inductively coupled mass spectrometry (ICP-MS) measured metals in diluted whole blood. ICP-MS is a validated technique for analyzing metals in biological media.

All data set metal analytes had the same detection limits. An imputed fill value was placed in the analyte results field for analytes below the lower limit of detection using the equation: *lower limit of detection divided by the square root of 2* [23].The NHANES Laboratory Procedures Manual describes specimen collection and processing in detail [24]. The National Center for Environmental Health (NCEH) of the CDC’s Division of Laboratory Sciences performed metal assays on whole blood samples for the NHANES 2007–2014. Blood metals were identified and quantified using the inductively coupled plasma mass spectrometry method No. ITB0001A.

### 2.3. Determining Allostatic Load Levels

This study’s AL was determined using physiological evaluations of 10 health indicators or biomarkers. The biomarkers included systolic blood pressure (SBP), diastolic blood pressure (DBP), total cholesterol (TC), high-density lipoprotein (HDL) cholesterol, glycosylated hemoglobin (HbA1c), albumin (Alb), triglycerides (TG), body mass index (BMI), creatinine clearance (CLCR), and C-reactive protein (CRP). Measures of AL were determined by calculating the cutoffs for various biomarkers based on their distribution within the database. All biomarkers were transformed into quartiles based on the data distribution. The top 25% of the distribution for each marker was designated as high risk for (1) C-reactive protein (CRP), (2) triglycerides (TG), (3) total cholesterol (TC), (4) systolic blood pressure (SBP), (5) diastolic blood pressure (DBP), (6) body mass index, and (7) glycosylated hemoglobin. For the other markers where high risk is determined by lower values, the bottom 25% of the distribution was used. These markers included (1) urinary albumin (Alb), (2) creatinine clearance (CLCR), and (3) high-density lipoprotein (HDL) cholesterol. High risk for each marker was assigned a value of 1, with low risk assigned a value of 0 to obtain a total AL index out of 10. An AL value greater than 3/10 was considered elevated, as indicated by the prior work of the team and others [2,25,26,27].

### 2.4. Data Analysis

We used BKMR with the hierarchical variable selection method due to highly correlated variables and collinearity in the datasets. We utilized the BKMR model in the R program using the R package (bkmr) to simulate the dataset. In this study, the model evaluated the impacts of mixtures or multipollutant exposures (e.g., PFAS and metals such as cadmium, cobalt, cesium, molybdenum, lead, etc.) by comparing the implementation of statistical and characteristics methods using the (kmbayes) function.

#### BKMR Modeling for Binary Outcomes

Combining data sources from various samples, including probability and nonprobability samples, is appropriate when using Bayesian inference. The use of Bayesian inference has various benefits. It first enables the estimation of complicated models and the quantification of uncertainty measurements.

The likelihood function can be used to analyze sample units based on probability.

As the probability sample size grows, it is primarily set up to give these units priority in the posterior calculations. Third, it enables posterior estimates to be more effective and efficient than estimates obtained from tiny probability-only samples, with less uncertainty [19,28].

We implemented kernel machine regression (KMR) for binary outcomes, as follows:

Φ − 1(P(Yi = 1)) = h(zi1, …, ziM) + βxi, I = 1, …, n

where h is a flexible function of the predictor variables zi1, ..., ziM, x is a vector of covariates (β is the corresponding vector of coefficients), and h is the cumulative distribution function (CDF) for the standard normal distribution. The outcome Yi is a binary (0/1) variable. Predictors z were the exposure variables, and h(.) was the exposure-response function. A kernel machine representation was used to model the function h in order to capture complex, non-additive, non-linear exposure-response interactions.

**Figure 1:**
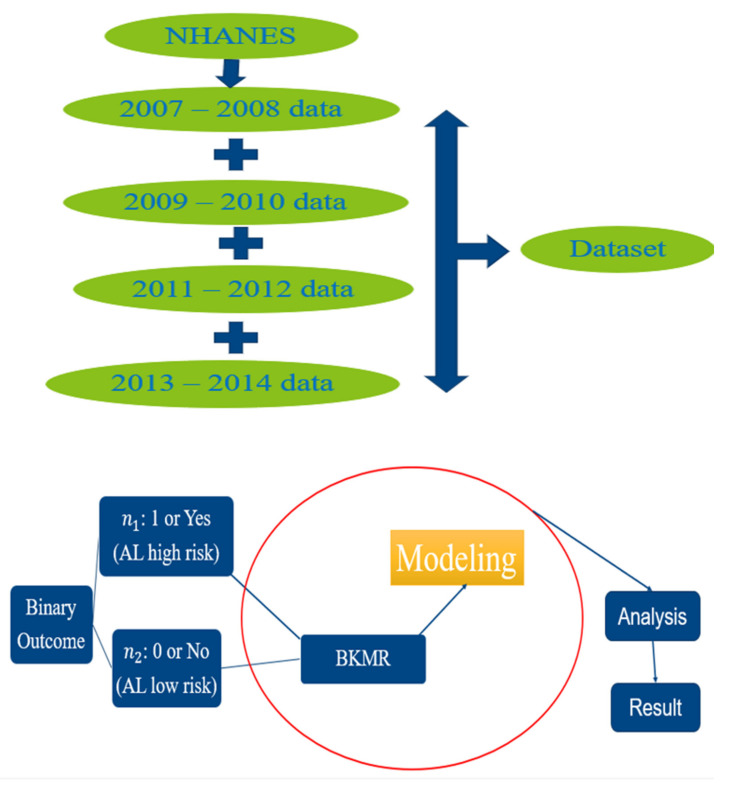
Flow chart for methodology.

The outcome variable in this study was AL. AL index values ≥ 3 were considered high risk, with values < 3 considered low risk. Those who were high risk were assigned a 1 in the dataset, while those who were low risk were assigned a 0. Binary outcomes were performed by applying the BKMR package using the probit model for convenience of computation and to overcome some of the issues that may arise in the dataset, such as collinearity under Bayesian inference [29]. Posterior inclusion probabilities (PIPs), which offer a gauge of the variable importance of each exposure, were extracted and plotted. All models were adjusted for sex, age, smoking, physical activity, ethnicity, occupation, income, alcohol consumption, education, birthplace, and time in the U.S. The analysis within this study was conducted using R software, version 4.1.2 (R Foundation for Statistical Computing, Vienna, Austria). A flow chart containing all the steps performed in the analysis can be found in Figure 1 below.

## 3. Results

Table 1 below provides the posterior inclusion probabilities (PIPs), which measure the percentage of the data that backs the inclusion of exposure or variable in the model. In other words, it quantifies the variable significance to be included within the model. The exposures to be included in the model were PFNA, PFUA, PFOA, PFHS, mercury, cesium, and molybdenum.

**Figure 2:**
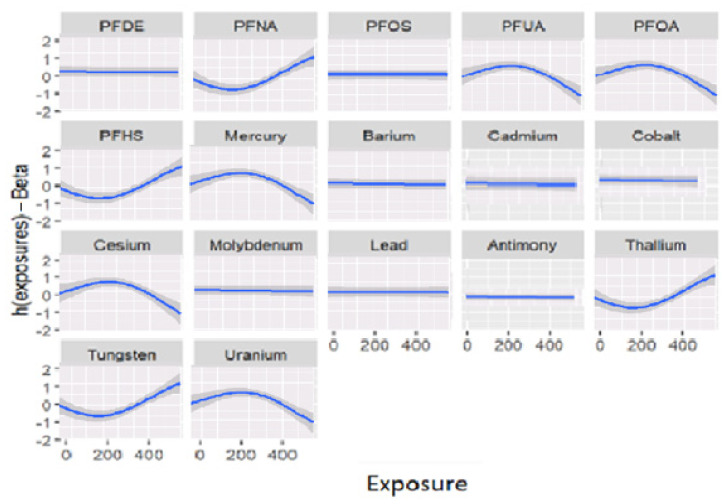
Univariate exposure-response functions and 95% confidence intervals for each metal and PFAS exposure, with others fixed at the median.

Figure 2 shows the association between the response variable and each individual exposure included in the model, which is known as the univariate relationship. The other exposures were fixed at their median values, and the covariates were fixed as constant. This figure shows that the association of some variables is not significant or has no association with the outcome. In other words, Figure 2 below shows the univariate independent-response association (each individual independent and dependent—AL association) by fixing the remaining exposures to their median, with the covariates being constant. The associations in Figure 2 present the relationship of exposures with responses when the model is adjusted for covariates (sex, age, smoking, physical activity, ethnicity, occupation, income, alcohol consumption, education, birthplace, and time in the U.S.). For instance, exposure to PFNA, PFUA, PFOA, PFHS, mercury, cesium, thallium, tungsten, and uranium are associated with AL, with some of these contaminants having sharper inclines, indicating different levels of exposure. Uphill on the graphs represents a higher level of exposure, and downhill shows lower levels of exposure. In other words, concentration values increase and decrease depending on the amount of exposure.

In Table 2, the PIPs with the highest values are explored using critical sociodemographic and behavioral variables. The six highest PIPs were molybdenum, cesium, mercury, PFNA, PFOA, and PFHS.

Table 3 explores mean AL levels by ethnicity and age group. This was performed to give context to the results. The results indicated that both ethnicity and age are significantly related to AL.

Table 4 explores the correlation between all the critical environmental exposures in this study. The results demonstrate that the strongest correlation exists between cesium and mercury.

## 4. Discussion

The main PFAS have extensive half-lives in humans and are physiologically and biologically persistent. The gap in the body of knowledge on the impact of environmental pollutants on stress and health is partly filled by attempting to understand the relationship between the cumulative physiological burden of stress (AL) and PFAS and metals [30]. This is especially true because stressors are constantly present in people’s lives, and the cumulative effect on health is apparent when resilience is lacking [30,31].

BKMR provides a way to address the potential multicollinearity among numerous PFAS and metal exposures, which cannot be resolved using traditional regression modeling. Based on a comprehensive analysis of the NHANES 2007–2014 data, we assessed the relationships between metal and PFAS exposures and AL among a nationally representative sample of adults. The study’s findings supported the main hypothesis, which stated that exposure to a combination of PFAS and metals is strongly linked to AL. This expands prior work by the team, which found that metals and PFAS are associated with AL using simpler modeling techniques [2,30].

In this study, combined exposure analyses of PFAS and metals showed a significant positive association between mixed PFAS and metal exposure and AL, to which cesium, molybdenum, PFHS, PFNA, and mercury contributed the most (PIP = 1, 1, 0.854, 0.824, and 0.807, respectively). In addition, the correlation between selected metals and PFAS (Table 4), with some negatively and others positively associated, suggests that the relationships between these factors are varied and require dynamic modeling techniques to capture the combined relationship appropriately. In the BKMR model, a substantial positive association between combined metal and PFAS exposure and AL existed for PFNA, PFUA, PFHS, thallium, and tungsten.

The univariate relationship between AL and each exposure in the model is depicted in Figure 2. All other exposures and covariates were held constant at their respective median values. The results demonstrated which variables, in combination, were not significantly associated with AL. These models were adjusted for confounding factors, and the associations between exposures and responses became clear. Exposures to PFNA, PFUA, PFOA, PFHS, mercury, cesium, thallium, tungsten, and uranium, to name a few, are all associated with AL; some of the graphs had steeper slopes than others, reflecting the fact that there were varying degrees of association between variables.

The molecular processes or toxicological pathways that underlie the relationships between human exposure to PFAS and metals and AL are not fully understood. The means by which exposure to PFAS and metals brings forth adverse health outcomes may be via AL. Simply put, AL may be the mediator between exposure to multiple contaminants and adverse health outcomes [2,32], such as heart disease, high blood pressure, metabolic syndrome, obesity, and arthritis [33].

Table 2 shows that the mean levels of the contaminants of interest varied by ethnicity; for example, Asians had high mean levels of molybdenum, cesium, and PFUA, with values of 65.9, 537, and 0.26, respectively. Blacks had higher mean mercury levels, and Whites had higher PFOA and PFHS levels than the other groups. These varied exposure levels by ethnicity speak to the variability of the contaminants of interest and the dynamism of exposure in various environments.

Within our results, compared to those of the White, Asian, and Hispanic ethnicities, non-Hispanic Blacks had greater rates of high AL. Table 2 and Table 3 demonstrate that across all age groups, high stressors in addition to lower levels of resilient behavior, such as physical activity, exist. This may play a role in adverse health outcomes driven by AL.

Understanding the social implications of AL may help explain some of the results of this study. For instance, many ethnic groups in the U.S. experience prejudice, face poor wage employment disproportionately, and are susceptible to chronic stress [34]. In the context of multiple environmental exposures, these factors may play a role in promoting AL. When this is intertwined with inadequate healthcare, the health burden on communities exposed to combinations of exposures and health outcomes is vast [34]. Non-Hispanic Whites in the US often have lower levels of AL than minority ethnic groups, as demonstrated in Table 3, across all age groups [35]. This may partly explain the lower disease burden within this group compared to the other groups.

Age is a critical variable in AL levels, with younger people typically having lower AL levels than older persons [36]. Our results, as shown in Table 3, confirm this. Continuous stressor exposure over the course of a lifetime can promote inflammation and oxidative stress, which can lead to physiological impairment and promote disease [37]. Among these is cardiovascular disease, the leading killer in the U.S. and in the world [38]. As people become older, their biological sensitivities to chronic stress vary, and the body’s physiological response system also changes naturally. As a result, biological regulation may deteriorate over time, which may result in an unhealthy physical state. This scenario has the potential to cause mortality over time, especially in elderly people [35].

The literature on AL by sex varies. Some research has shown that AL levels are often lower in men who hold professional positions, such as managers and directors. On the contrary, Rogers et al. reported that men with higher levels of education are likely to have higher AL [39]. According to several studies, women who obtained higher levels of education and simultaneously held professional jobs as managers had a higher prevalence of AL [40]. People who experience continual stress due to issues such as unemployment and poverty are more likely to engage in excessive drinking, smoking, and eating, which leads to obesity, poor sleep, and, of course, increased AL [41]; our results in Table 2 and Table 3 support this.

According to a study by Petrovic et al., drinking, smoking, and eating too much sodium were all associated with a higher risk of developing AL. Meanwhile, physical activity and a vegetarian diet were linked to a reduction in AL [40]; our results in Table 2 support these findings.

Very few laboratory studies have examined combined exposure to PFAS and metals. Therefore, future experimental and human investigations are required to further corroborate our findings and to investigate the probable mechanism for the health impacts of PFAS and metal exposure on AL, given the dearth of laboratory data and the cross-sectional design of our study. The limitation of this design means that temporality cannot be inferred. A longitudinal study would offer better insight into these exposures and health outcomes.

## 5. Conclusions

PFAS and other toxicants, such as metals, interact in the human body to produce AL. The mixture of PFAS and metals is critical to understand, as they may, in combination, bring forth adverse health outcomes via AL. When PFAS are found in the body alongside metals, our results indicate that their combined toxicity needs to be considered, with cesium, molybdenum, mercury, PFHS, and PFNA especially being of concern. More research is required into this matter. Research into the levels of exposure to multiple pollutants required to bring about AL must be explored if we are to gain an understanding of the real-world mixture concentrations that bring forth disease. This is of paramount significance for at-risk communities because their members lack the resources to effectively manage stress and/or avoid exposure to environmental contaminants.

## Figures and Tables

**Table 1:** Posterior inclusion probability (PIP) and comparison of four models (BKMR, Oracle, Linear, and True) of overall health effects on the response variable (AL) by exposures.

			**Overall Effects**			
			Models			
			BKMR ^1^	Oracle ^2^	Linear ^3^	TRUE ^4^
			0.680	−0.539	−0.572	−0.843
			Individual effect			
			Models			
variable #	variable	PIP ^5^	BKMR	Oracle	Linear	TRUE
1	PFDE	0.700	0.068	0.153	−0.693	−1.784
2	PFNA	0.824	0.023	0.239	0.124	0.264
3	PFOS	0.651	0.087	−0.131	0.032	0.059
4	PFUA	0.795	0.019	0.098	0.235	−0.324
5	PFOA	0.754	0.033	0.955	0.015	−0.021
6	PFHS	0.854	0.115	−0.169	−0.037	−0.068
7	Mercury	0.807	0.022	−0.278	−0.084	−0.144
8	Barium	0.719	0.014	0.427	0.033	0.054
9	Cadmium	0.727	0.390	0.356	0.114	0.199
10	Cobalt	0.706	0.022	−0.038	−0.169	−0.273
11	Cesium	1.000	0.350	0.101	0.003	0.008
12	Molybdenum	1.000	0.238	−0.386	0.004	0.006
13	Lead	0.674	0.035	0.736	−0.008	−0.036
14	Antimony	0.701	0.203	−0.258	0.343	0.543
15	Thallium	0.749	0.044	−0.163	0.419	0.663
16	Tungsten	0.762	0.012	−0.195	−0.349	−0.689
17	Uranium	0.723	0.016	−0.413	4.204	7.213

Note: ^1^ Bayesian kernel machine regression (BKMR); ^2^ Oracle model that uses glm (generalized linear model); ^3^ Linear model; ^4^ True model using all variables with no adjustment; ^5^ PIP (posterior inclusion probability), which quantifies the importance of the variable in variable selection.

**Table 2:** Metals (molybdenum, cesium, and mercury) and PFAS (PFNA, PFOA, and PFHS) means by critical study variables.

	**Metals and PFAS**					
**Variable**	**Molybdenum**	**Cesium**	**Mercury**	**PFNA**	**PFOA**	**PFHS**
**Activities**	**Mean**					
1 day	57.80	5.08	0.59	0.14	0.98	0.80
2 days	76.70	4.72	0.62	0.13	0.89	0.77
3 days	55.70	5.05	0.57	0.13	0.85	0.70
4 days	45.30	5.20	1.33	0.12	0.84	0.72
5 days	60.40	4.26	0.57	0.13	0.90	0.79
6 days	40.10	3.56	0.44	0.13	1.00	0.82
7 days	57.40	3.60	0.63	0.14	0.86	0.67
Smoke						
yes	51.70	4.95	0.56	0.13	0.93	0.78
no	61.00	5.03	0.64	0.14	0.81	0.67
**AL**						
high	67.54	5.35	0.60	0.13	0.83	0.73
low	49.45	4.73	0.61	0.13	0.88	0.71
Ethnicity						
Mexican	59.10	5.07	0.58	0.09	0.78	0.59
Black	57.70	4.78	0.66	0.14	0.84	0.72
White	50.20	4.89	0.54	0.12	0.97	0.82
Hispanic	61.80	5.19	0.43	0.11	0.80	0.59
Other and Asian	65.90	5.37	0.60	0.26	0.65	0.60
Sex						
Female	54.30	4.76	0.63	0.12	0.76	0.56
Male	59.90	5.23	0.58	0.14	0.97	0.88

**Table 3:** AL means for ethnicities by age group.

	**Age Groups**		
	20 to 39	40 to 59	60 and older
Ethnicity	AL mean		
Mexican	2.9	3.49	3.58
Black	3.32	3.92	3.83
White	2.66	3.26	3.37
Hispanic	2.69	3.47	3.64
Other and Asian	2.63	3.08	3.13

**Table 4:** Correlation between metals and PFAS variables.

**Metals and PFAS**						
	**PFNA**	**PFOA**	**PFHS**	**Cesium**	**Molybdenum**	**Mercury**
**PFNA**	1.000	0.124	0.028	0.042	−0.006	0.064
**PFOA**	0.124	1.000	0.327	0.003	0.032	−0.032
**PFHS**	0.028	0.327	1.000	0.029	−0.013	−0.069
**Cesium**	0.042	0.003	0.029	1.000	0.320	0.438
**Molybdenum**	−0.006	0.032	−0.013	0.320	1.000	0.221
**Mercury**	0.064	−0.032	−0.069	0.438	0.221	1.000

## Data Availability

The NHANES dataset is publicly available online, accessible at cdc.gov/nchs/nhanes/index.htm (accessed on 12 January 2023).
